# Editorial: Recent advances in biotechnological applications of microbial secondary metabolites

**DOI:** 10.3389/fmicb.2024.1525146

**Published:** 2024-12-09

**Authors:** Ragini Bodade, Ajay Kumar, Vijay K. Sharma, Paola Angelini

**Affiliations:** ^1^Life Science Division, Institute of Advanced Study in Science and Technology, Vigyan Path, Guwahati, Assam, India; ^2^Amity Institute of Biotechnology, Amity University, Noida, India; ^3^Agricultural Research Organization, The Volcani Center, Rishon LeZion, Israel; ^4^Department of Chemistry, Biology and Biotechnology, University of Perugia, Perugia, Italy

**Keywords:** secondary metabolites, microbial strains, fermentation, human health, microbial metabolites

Secondary metabolites are low molecular weight organic compounds synthesized by microbes during the late growth stage. Although secondary metabolites do not play a direct role in growth, but they have important ecological functions for microbes. The synthesis and production of microbial secondary metabolites are influenced by various factors such as growth conditions, media composition, feedback regulation and enzyme activation/inductions. Moreover, secondary metabolites are derived from primary metabolites, produced under the control of biosynthetic gene clusters that are regulated by tRNA, low molecular weight metabolites and gene products formed during post-exponential development. Recent advancements in biotechnological applications of microbial secondary metabolites have highlighted their potential uses in various sectors including sustainable agriculture, environment contamination and human health management.

The Research Topic “*Recent advances in biotechnological applications of microbial secondary metabolites*” has compiled 10 intriguing papers on production, characterization, regulation, and diverse applications of microbial secondary metabolites.

For example Narsing Rao et al. reported about exopolysaccharides secreted by the thermophilic strain *Bacillus paralicheniformis* CamBx3, which exhibit strong antioxidant and β-glucosidase inhibitory activities and form viscoelastic gels at acidic pH levels. The genome analysis of the strain provided information about EPS gene clusters.

Li et al. characterized four new polyketides and seven known compounds from fermented extracts of *Paecilomyces gunnii* YMF1.00003. Among different compounds 3β-hydroxy-7α-methoxy-5α,6α-epoxy-8(14),22*E*-dien-ergosta showed potent cytotoxic activity against five tumor cell lines, while the compounds gunniiol A, 7*R*-[[4*R*,5*S*-dihydroxy-1-oxo-2*E*-hexen-1-yl]oxy]-4*S*-hydroxy-2*E*-octenoicacid and (3*R*,5*R*)-3-hydroxy-5-decanolide exhibited protein kinase Cα inhibitory activity. The potential of extracted secondary metabolites can be significantly enhanced by manipulating specific transcription factors.

Kaliaperumal et al. discovered seven compounds from fermentation extracts of *Penicillium verruculosum* (XWSO1F60), endophytes of *Spongia officinalis*. These compounds were averufin, aspergilol-A, sulochrin, monomethyl sulochrin, methyl emodin, citreorosein, and diorcinol. Notably, averufin showed anticancer activity against myeloid leukemia HL60 cell lines at IC_50_ concentration of 1.00 μM compared to the standard taxol (0.002 μM) by *in vitro* assay. Moreover, virtual computational molecular docking studies confirmed the considerable binding between averufin and HL60 antigens.

Wang et al. investigated the cyclosporin A (CsA) production from *Tolypocladium inflatum* and the effect of varying concentrations of fructose and sucrose on its mycelium growth. They observed that high levels of fructose in the medium resulted in enhanced CsA production. This study provides valuable information about potential candidate genes that could be modified through metabolic engineering to create strains for higher CsA yield.

Shahbaz et al. reported the capabilities of probiotics in improving meat quality. This study reported the isolation of four lactobacilli strains from chicken gut. All strains showed strong resistance to salt and bile salts, as well as good viability and adherence ability to chicken ileum epithelial cells. *Lactobacillus delbrueckii* PUPro2 displayed a faster growth rate compared to the other strains. Furthermore, all the strains exhibited antagonistic behavior against the tested pathogens, cholesterol assimilation capabilities, and γ-hemolysis. The study revealed that these bacteria have the potential to enhance chicken growth and improve meat quality.

Lu et al. isolated a salt-tolerant rhizobacteria, *Micromonospora profundi* TRM 95458, from the rhizosphere of chickpea plant (*Cicer arietinum* L.) and extracted a novel osmotic compound named 2-(2-(2,3-dihydroxypropoxy)-2-oxoethyl) amino) benzoic acid (ABAGG) from its fermentation broth. The strain *M. profundi* TRM 95458 showed the capability to convert glycerol into ABAGG. Additionally, accumulation of ABAGG was found to depend on the concentration of glycerol and glycine in the medium. The study suggests the accumulation of ABAGG in the *M. profundi* TRM 95458 enables the strain to thrive in saline-alkaline environments.

Shi et al. investigated the antagonistic activity of *Bacillus velezensis* BHZ-29, which has shown the ability to combat *Verticillium dahliae*, a plant pathogen responsible for *Verticillium* wilt in cotton. This strain exhibited a high potential for inhibition, achieving a disease control ability of 93.8% in treated cotton. The strain increases the activities of peroxidase and superoxide dismutase, indicating that it employs antibiosis while also inducing resistance, thereby showcasing its promising role in agricultural applications.

Kim et al. conducted a comparative genomics study to analyze secondary metabolite gene clusters in 366 different *Burkholderia* species. Their research revealed distinct patterns of these gene clusters within different groups of *Burkholderia* species and examined the relationships between species and metabolite synthesis (polyketide synthase, terpene, and siderophore) through network analysis. The study highlighted similar patterns of siderophore gene clusters among various species, providing insights into their species-specific mechanisms of environmental adaptation.

Yuan et al. reported that microalgae accumulate fatty acids and astaxanthin in response to abiotic stress. Their research emphasized the significant role of NADPH oxidase-derived reactive oxygen species in accumulation of fatty acids and astaxanthin in *Chromochloris zofingiensis* under high-salinity, nitrogen and phosphorus stress. They identified 1,445 shared differentially expressed genes through transcriptome analysis. Additionally, enrichment analysis suggested the importance of biotin, betalain, thiamine, and glucosinolate in stress responses. The heat map illustrated that diphenyleneiodonium notably suppressed gene expression in the fatty acid and carotenoid biosynthesis pathways.

Xu et al. examined the dynamics of microbial flora associated with musk during its secretion period using a metagenomics approach. Male musk deer secrete a malodorous liquid from their musk glands, which, upon fermentation, turns into a blackish-brown solid in the musk pod, resulting in the distinct musk scent. The serum testosterone level, chemical composition, and microbiota of musk exhibit dynamic changes during its secretion. GC-MS analysis of natural musk revealed maximum production of 3-methyl cyclopentadecanone followed by 3a-hydroxy-5b-androstan-17-one and cholesterol. Studies of mascon using 16S rRNA sequencing from different stages of animals showed *Actinobacteria, Firmicutes*, and *Proteobacteria* were the dominant bacterial phyla throughout the musk secretion cycle. Moreover, *Pseudomonas* and *Corynebacterium* were identified as the biomarkers during the vigorous musk secretion period, while *Clostridium* appeared in the late period. *Actinobacteria* and *Corynebacterium* were predicted to be involved in the synthesis of muscone and etiocholanone during musk secretion.

The studies presented in this Research Topic highlight the synthesis, structural characterization and diverse potential applications of secondary metabolites across various fields. They emphasize the relevance of these compounds in environmental sustainability, human health, and animal welfare. It is our hope that this Research Topic offers valuable scientific insight into microbial secondary metabolites while addressing critical knowledge gaps in the field.

